# Documenting Families: Paper-Work in Family Display among Planned Single Father Families

**DOI:** 10.1177/00380385211073238

**Published:** 2022-03-08

**Authors:** Sophie Zadeh, Vasanti Jadva, Susan Golombok

**Affiliations:** University College London, UK; University of Cambridge, UK; University College London, UK; University of Cambridge, UK

**Keywords:** documents, family display, paperwork, single fathers, single fathers by choice

## Abstract

This article extends existing sociological scholarship on doing and displaying family by developing the concept of documenting families. We suggest that documenting is conceptually rich insofar as it showcases the relationship, and tensions, between institutional practices and individual experiences of family display. Drawing on our research with men who became parents without partners, we argue that the process of documenting family is made especially evident in studies of what Finch originally referred to as ‘non-conventional’ family relationships. We explain that documenting sheds light not only on the official and unofficial means through which families are recognised on paper, but also on family practices as work – in this case paper-work – that involves negotiation between different social actors who are generally unequal in terms of their authority and agency to impose situational meaning.

## Introduction

‘Doing family’ and ‘displaying family’, respectively introduced by Morgan (1996) and Finch (2007), are key analytical tools in the family sociologist’s kit. Each foundational to a now vast body of scholarship that views families as sets of practices, the former highlighted the routine, everyday and taken-for-granted doing of family, while the latter emphasised that families are done within social contexts – and as such, they are *displayed* by, with and to social actors. According to Finch (2007), display as an analytical tool concerns the interrelated presentation and recognition of certain actions as ‘family-like’. Display, she argued, is characterised by varying degrees of intensity across different circumstances and over time, leading the concept to have since been especially employed and interrogated by scholars researching non-normative (Finch’s (2007) ‘non-conventional’) family relationships. These relationships differ from those in ‘normative families’ with two heterosexual, cisgender parents who, within the context of a monogamous romantic relationship, have had children to whom it is assumed they are genetically related.

In this article, we focus on planned single father families: families headed by men who decided to become parents without partners, most often because they were not in romantic relationships when they decided to parent. Single father families comprise 20% of all single parent families in the USA (US Census Bureau, 2019), while the comparable figure for the UK is 10% (Office for National Statistics, 2021), although such statistics do not alone indicate how prevalent the various pathways to single fatherhood are. It is likely that men taking a planned route to single fatherhood are in the minority, although this population also appears to be increasing year on year (Brilliant Beginnings, 2021). In the existing sociological literature, little has been said about men who become parents without partners. Much, however, has been written about the ‘new’ or ‘involved’ father (Dermott, 2008), whose role is thought to have undergone significant cultural and conceptual change, such that it now extends beyond the financial provision traditionally thought of as men’s ‘work’ within families. In spite of supposed societal shifts in conceptualising fatherhood, researchers have also identified that fathering in fact largely reflects continuities of practice, primarily in the gendered division of labour within ‘conventional’ family relationships, often despite couples’ pre-parenthood intentions (Miller, 2010), and the resources available to them (O’Brien and Shemlit, 2003). The reasons for this have been the source of much scholarly debate (Gatrell and Dermott, 2018), with a particular emphasis on policy contexts that continue to reinforce traditional gendered divisions around work and care (O’Brien and Wall, 2017).

The existing legislation relating to planned single fatherhood – specifically through surrogacy and adoption – varies, with some contexts (e.g. the UK, parts of the USA) being more progressive than are others (e.g. France, Spain). Following a landmark ruling in the UK family court in January 2019, it is now possible for single people in Britain to apply for parental orders following the birth of their children through surrogacy, and thus become their legal parents (Krajewska and Cahill-O’Callaghan, 2020). Single men in the UK can also become parents through adoption. Regardless, legal permissibility does not necessarily equate to social acceptability, either in general (Maya and Adital, 2021), and/or among those who determine access to key services (Hicks, 2006).

Throughout this article, we describe planned single father families as ‘non-conventional’ and/or ‘non-normative’ to reflect the sense in which they are different, structurally speaking, to ‘the family’ as it is socially sanctioned. Perhaps unsurprising given Finch’s (2007) original assertion that display may be especially required of ‘non-conventional’ relationships, the existing literature using this concept has mostly examined display in families headed by same-sex couples (Almack, 2008; Dempsey and Lindsay, 2018; Frank et al., 2019) as well as in trans* parent families (Zadeh et al., 2021). This research has focused on naming (through surnames and parental designations) and family narratives, forms of display that Finch (2007, 2008) herself recognised as important. Yet questions have also been raised about display as it relates to such families, in particular those headed by same-sex couples. Some scholars have argued that the original concept does not adequately address the ways in which family practices inevitably relate to dominant representations of ‘the family’ (Heaphy, 2011) or the fact that some families are prevented from successful display by virtue of their non-normative composition (Almack, 2008; Gabb, 2011). Other authors have stressed that while some families who lack social recognition may choose to engage in display work, other families may reject the very idea, thus suggesting that display relates to social norms about families that may or may not be acceptable to their members (Almack, 2008; Ryan-Flood, 2011; Short, 2011). Most recently, the notion of display has been theorised, to encompass these different approaches, as reflexive in nature (Mamali and Stevens, 2020).

In responding to some of these ideas, Finch (2011) reiterated the importance of the actor–audience nexus, suggesting that focusing on the audience (e.g. to whom displays are done and with what effects) had led scholars to be overly critical of the concept of display without adequate empirical exploration. At the same time, she conceded that family display might be of insufficient analytical rigour to explain those ‘attempts to impose meaning of the situation [that] are overwhelmed by broader, established cultural meanings of family relationships’ (Finch, 2011: 205). The concept of documenting developed in this article is a means of theorising the relationship between social structure (e.g. as embedded in legislation) and agency (e.g. the activities of family members) in family display, and therefore may overcome the possible shortcomings of display that Finch (2011) previously identified.

Documenting refers to the official and unofficial processes of producing material to display and do family, and is a concept that is intended to contain within itself the tensions between institutional (‘official’) systems and individual (‘unofficial’) practices of family display among non-normative families, such as those headed by men without partners. Indeed, we describe such families as ‘non-normative’ to bring into focus the role of what Finch (2011) referred to as the ‘official bureaucratic audience’ in displaying family. Alongside this positioning, however, we retain Morgan, Finch and others’ emphasis on the ordinary, everyday and fluid meanings of family (Morgan, 2011), and use Morgan’s (2004) distinction between fatherhood, fathering and fathers to acknowledge that all such single fathers do not ‘do’ or ‘display’ family in the same way. As will be shown, display involves *negotiation* (Finch and Mason, 1993: 59) – a process that has been acknowledged as ‘never entirely open-ended and sometimes … tightly constrained’. In such negotiations, which mostly take place between social actors who are unequal in their authority and agency to impose situational meaning, documents are central.

Indeed, like family narratives (Finch, 2007), documenting refers to both actions (e.g. to document) and products (e.g. documents). While some products, such as official birth certification, are said to span over 500 years (Brumberg et al., 2012), others, such as online records of individual family histories, are relatively recent (Davison, 2009). Yet scholarly interest in the fact that families are in some sense documented is itself not new. From written census records to collections of family photographs, social scientists have long studied both ‘official’ and ‘unofficial’ records of family life, what these might tell us about how family lives are lived, and what they reveal about familial identities (Carsten, 2000; Kramer, 2011; Woodham et al., 2017). However, despite the example of family photographs originally given by Finch (2007), there has been limited focus on the relationship between such records of family life and the interactionist perspective within family sociology. Notable exceptions are Smart’s (2007) *Personal Life*, in which the role of documents such as adoption records, photographs, personal diaries, marriage certificates and written correspondence in doing family was highlighted using a conceptual framework complementary to Morgan’s (1996) family practices; and Roberts et al.’s (2017) research on commercial ultrasound scans, the sharing of which was explicitly theorised as a form of family display.

‘Human documents’ (Blumer, 1979) or ‘documents of life’ (Plummer, 2000) – biographies, diaries, letters and photographs, among others – have of course elsewhere been recognised as sociologically valuable, although it has been said that scholars have generally focused *either* on documents’ content, *or* their uses and functions (Prior, 2008). The latter refer both to how individual actors use documents, and how they function in social interaction and organisation. The idea that texts fundamentally participate in social relations (Smith, 1993) is clearly relevant for those working with notions of doing and displaying family. Yet while the role of birth certificates (Short, 2011), civil partnership legislation (Gabb, 2011; Ryan-Flood, 2011) and ‘official’ audiences including adoption panels and social workers (Haynes and Dermott, 2011) in family display has been noted, there has yet to be any substantial theoretical engagement with documenting within this literature. This is perhaps surprising given that research on ‘non-conventional’ relationships often highlights the role of official documents in regulating family lives and experiences (Gibson, 2016; Nordqvist, 2012), particularly when family formation involves several countries, as in transnational surrogacy (Courduriès, 2018; Deomampo, 2015; Jadva et al., 2021). The significance of official documents has also been articulated in other areas of social scientific inquiry, including in studies of citizenship and national identity (Makarychev and Yatsyk, 2017) and gender identity (Currah and Moore, 2009), where the absence of such documentation has been shown to relate to experiences of marginalisation across different social domains (Chereni, 2017; Ryan, 2020).

In this article, we focus mainly on the uses and functions of documents, that is, how they are used, and what they are *for*. Following Smith (1993), we examine how documents function in episodes of social interaction. In so doing, however, we inevitably also consider their content. We take documenting to mean the official and unofficial processes of producing material to display and do family, and we are especially concerned with documenting as a process that is undertaken by family members rather than by institutional actors, although the latter inevitably play a role (sometimes as co-actors, and sometimes as audience).

## The Study

This article draws upon an international, multidisciplinary study, which began in 2018, of planned single father families. It focuses on insights gained from in-depth individual interviews with 18 fathers. When interviewed, fathers were living in Australia, France, Spain, Sweden, Switzerland, the UK and the USA. Fourteen fathers had used a gestational surrogate, mostly in transnational surrogacy arrangements, and four had become parents through domestic adoption. Fathers had between one and six children, ranging in age from four months to 28 years. All but three had children under the age of 10, and all had children currently living with them. Although they each had in common having planned to become single fathers, one father also had a child from a previous relationship, and two fathers were now in relationships. In terms of sexual orientation, 16 fathers were gay, one was asexual and one was heterosexual. Among those fathers who provided further demographic information, 13 described their ethnic background as white, two as mixed, and two as other (using the UK Office for National Statistics classification). In terms of education, six fathers had master’s degrees, four had professional degrees, two had doctorates, two had undergraduate degrees, two had completed college and one had completed high school. They were recruited to the study with support from Brilliant Beginnings, Cafcass, Circle Surrogacy, Growing Families, and Family Equality, and through snowballing. The study received ethical approval from the Cambridge Psychology Research Ethics Committee.

Interviews were mostly conducted by the first author, either in person, over Skype or by telephone, given the study’s international nature. Each interview comprised the Parent Development Interview (Slade et al., 2005), designed to assess parents’ representations of themselves as parents, their children and their relationships with them, followed by a series of open-ended questions about experiences both prior to, and once having become, parents (including questions about any legal and practical challenges). Interviews ranged from 55 to 168 minutes (average length 108 minutes), and were transcribed verbatim, with identifying information redacted. We analysed all parts of the interview inductively. Transcripts were read, re-read and open-coded, at which point it became clear that documenting was key to fathers’ accounts. Indeed, the main way in which fathers ‘met’ institutions was in relation to documentation, and the extent and depth of information they provided about this was striking. Transcripts were therefore re-read and re-coded with this focus in mind, and an initial analysis formed using Finch’s (2007) concept of display as an anchor. This analysis was further honed during the process of writing, leading us to ultimately identify three related – but conceptually distinct – uses of documents. Following Smart (2007), the interview extracts below have been chosen not simply to evidence our analyses, but are deployed evocatively, to encourage further discussion on family display and the role of documenting in it. Each father has been given a pseudonym.

## Documenting as Paper-Work

Fathers generally described documents in ways that emphasised the *paper-work* required of them to present and have their families legally, institutionally and socially sanctioned. In the majority of our interviews, the official, mandatory processes – and institutional barriers – involved in documenting family were highlighted. The concept of paper-work is used to signify that documenting in relation to these constraints represented for many fathers a form of emotion work (Hochschild, 1979) that generated an unspoken sense of fatigue and/or frustration.

Our analysis showed documenting to serve several functions: to authenticate family relationships; to enable family practices; and to create and sustain family legacies. These functions each reflect complex relationships between family lives and the institutional barriers and social constraints within which they are lived. First, documenting as authenticating primarily describes the paper-work that fathers undertook to gain legal and institutional recognition of their families. Institutional parameters not only determined the often complicated and lengthy processes by which legal parental status was granted, but also created the contextual boundaries within which certain documents were deemed of value and relevance. Within such boundaries, few instances of explicit resistance to the normative assumptions about families embedded within documents (i.e. on official forms) were identified. Second, documenting as enabling family practices highlights the paper-work that served to enable family-like courses of action: in other words, that which could make possible fathers’ doing of family. Examples include interactions with different audiences (e.g. government officials, children’s doctors), and they showcase the different forms of negotiation undertaken by fathers in relation to wider norms (e.g. the explicit, implicit, and silent). Third, documenting to create and sustain family legacies extends Finch’s (2007) insights on narratives as a form of display, highlighting fathers’ use of documents as both testimonies of family life (e.g. for the general public) and records of family memories (e.g. for their children). As will be shown, these examples of documenting also evidence complex patterns in fathers’ displays that attest to the everyday nature of paper-work.

### Documenting as Authenticating Family Relationships

Fathers generally described documenting as a bureaucratic process to authenticate family relationships that began once they had decided to pursue parenthood and continued once they were parenting. These included instances of paper-work they described as either not required of other parents or as having taken on a specific character for them. Joseph, a father of a teenager with whom he lived in the USA, explained:

You know, the joke I always tell is that for a lot of families, having kids is something fun … but because I had to fill out all these papers over and over and over and over again, [for me] it was work … I was living in [US state] at the time, and went through their parenting classes, filled out all of the paperwork, did the physical exam, finances, five letters of recommendation, and then they did not approve my home study. It sat there in the office for a full year … then they said, ‘Oh, it’s been a year, you need to fill out all of that paperwork again.’

Later juxtaposing his experience with ‘straight couples’ he knew to have been rapidly approved as adopters, Joseph’s account reveals the lengthy – and cyclical – process required to display to an official audience that his family would ‘work’. This was also true of fathers living in other countries. One interviewee, Oscar, was based in Sweden, where surrogacy is legal but not practised in licensed clinics, and official documents produced in other countries that grant parental status in transnational surrogacy arrangements are not legally recognised (Arvidsson et al., 2019). Another, Luis, was in Spain, where surrogacy is illegal, and applications to register the births of children born through transnational surrogacy have been refused (Blanco, 2018). Their accounts underscore the extensive paperwork involved in gaining legal recognition of their families.

We had to go together to the [Swedish] embassy as well, so she could sign away parental rights to me because according to Swedish laws she had all the parental rights. Well, first she had to sign at the embassy, then we got a temporary passport for them, so we flew back to Sweden, then I had to sue her in court for custody because she still had official custody, that’s how it works in Sweden. Although she agreed the whole time I still had to sue her, and they had to contact her and check again if she agreed to give me parental rights, and she did, and I was awarded … but the whole process took about four months. I was very nervous during that time. (Oscar)

I’m still waiting for the Spanish birth certificate and this is slow … To register her the Spanish consulate want me to show a DNA test, which is something that’s not requested to anyone, only it’s requested to me because I did surrogacy, so they want to see that that’s my child, that it’s not human trafficking with another child that I found somewhere or something like this. Yeah, that for me is a legal challenge and it’s completely unnecessary. (Luis)

These examples evidence that the display of family relationships may be driven by specific legislative contexts that each require different types of ‘work’. Fathers’ descriptions of the process of obtaining official documents – as unnecessary and a source of anxiety – also illustrate the fact that documenting involves significant emotion work (Hochschild, 1979). Reflecting this, John, in the UK, referred several times in his interview to the fact that his son was ‘not legally mine’. In so doing, he highlighted the relationship between the institutional means through which he was required to document his family, and his feelings about his status as a parent.

John’s case is also illustrative of the fact that some fathers had undertaken additional paper-work in anticipation of their family display being called into question by others. We interviewed John after legislative changes in the UK to allow single fathers legal parentage of their children born through surrogacy had been ratified, but before they were brought into effect.

I’d spoken to my doctor’s surgery before … before I went out, letting them know that yes, I was having a son through surrogacy, he’d be coming back, and from the airport we actually went to the doctors and registered him and they sorted out his NHS number for him, so … everything, you know, from the kind of official side has actually been fine. It’s just knowing that legally he’s – on all systems, he’s not legally mine. (John)

John also explained that he returned to the UK with his son having ‘made certain I’d got all the legal surrogacy documentation with me’. In fact, several fathers described physically carrying such documents on their person, especially during international travel, and especially when children were newborns or infants. Joseph described, ‘When she was much younger, you know, baby, toddler, they recommended that we carried [the adoption decree] with [us]’, and Adam explained that, ‘You’re a man with a child, which raises suspicion at the airport, so you then have to have … legal confirmation [about] how you did it and that you’re going to apply for citizenship.’ These examples demonstrate not only that fathers understood the authenticating potential of documents in interaction, but also the fact that such interactions were likely to reflect normative ideas about the gendered division of labour in families that are embedded across policy contexts, and render fathers travelling alone with children as ‘suspicious’.

Fathers mostly described documenting involving bureaucratic systems in ways that reflected feelings of a lack of agency over the process (‘filling out the passport form and stuff you have to give … information … that you wouldn’t really want to talk about with everyone, but you have to do that’ (Alex)). However, other instances of documenting in institutional settings were described in terms that would suggest them to be purposeful. Such instances notably involved fathers explicitly resisting normative ideas about family relationships that are often embedded within official paperwork (Gibson, 2016). For instance, Geoff described:

In some things like school settings, a lot of things are still designed for mums as primary, so some forms I’ve had to strike out mum to say dad or father, because there wasn’t even the option, it was just assumed … [I know] that I’m not the only … we’re not the only family in that situation but it would be nicer to have a form that actually is more inclusive.

Geoff’s suggestion that institutional inclusivity within the school setting would be ‘nicer’ is arguably at odds with the accounts of ‘nervousness’ from fathers who described anticipating the receipt of documents that would grant them legal parental status. It is clear from such fathers’ accounts that the use of documents to authenticate family relationships is for them a necessary form of display that involves ‘unnecessary’ work. Given institutional constraints, this type of display work does not neatly correspond to existing analytical categories developed in research on ‘non-conventional’ family displays in social rituals (e.g. marriage rites; Mamali and Stevens, 2020). Men who become parents without partners who seek recognition of their families – at least of a legal kind – are generally unable to determine how they relate to wider norms in this process (i.e. how they present on legal documents). Rather, they are subject to institutional practices that amount to what Gibson (2016) has termed ‘systemic gatekeeping’, and the work of displaying family is thus both time-consuming and emotionally burdensome for them.

### Documenting as Enabling Family Practices

Above, we showed that John and Adam used official documents in interactions with institutional actors who raised questions about their families. Relatedly, fathers also described documenting in terms that would suggest that it is a process related to doing family. These displays were with different actors and audiences with varying degrees of authority and agency, including government officials, children’s doctors, healthcare visitors and airport security staff. In these cases, documenting appeared as a means that fathers saw as enabling them to do – or be prohibited from doing – ‘family-like’ things, such as receive state support for parents, travel, or make medical decisions for their children:

The whole process took about four months, so it took a while to, to get it cleared, and during that time there was no official support, I wouldn’t get any. We get a monthly payment now for children for example … but I got that after four months … but it could take longer … then they have no guardians for the children, I mean you have no one, you can’t travel with them, you can’t, can’t really do anything … luckily that didn’t happen. (Oscar)

On his medical records it’s down that I don’t have the right to make medical decisions for him, so when you go in for shots you’re told, ‘Oh well you don’t have the right to make this decision.’ You’re going, ‘Yeah, you’re the doctor, he’s 10 months, do what you need to do’, you know, and you know that they’ll do what is in his best interests … but … I would like … because I can’t do anything about it you kind of put it to the back of your mind that there’s no point getting angry, upset or anything else about this. It’s what can I do that’s constructive? – And beyond that you just sit and wait it out knowing that hopefully January I get to apply, July he’ll then legally be mine. (John)

In his interview, Oscar emphasised that his wait to obtain official documentation – which made him ‘very nervous’ – could have nevertheless been longer and more stressful, owing to ‘case-by-case’ legal processes in Sweden (Arvidsson et al., 2019). John also expressed ambivalence about documenting as enabling him to engage in everyday family practices. In another interview, Paul, in the UK, described his discomfort that paperwork completed by health visitors included potentially damaging misinformation about his child and their relationship. Taken together, these examples highlight that the content and meaning of documents may not be created – or endorsed – by fathers themselves (Gibson, 2016).

Such examples also bring into focus the ways in which documents and their functions are actively negotiated in specific interactions (such as those that take place at the doctor’s surgery). Other interactions took place at airports, and with healthcare visitors:

Things like when we travel internationally, on the kids’ birth certificate where it says mum, it literally says just … just a space intentionally left blank, and so I get the questions of, ‘Who is their mum?’ You know. ‘Well they don’t have a mum.’ And then … depending on the logic of the person, ‘Well everybody has a mum.’ Well yeah, they have somebody that gave birth to them, but they do not have a legal parent, you know, and so those types of things come about. (Andrew)

They created an issue with the, the health visitor because their system would only actually allow you … the health visitor has actually pointed out that, ‘Well no, we need the mother’s details’, you know, ‘We can’t register you without them’, and I went, ‘Well I’m sorry but no, there is no mother on the birth certificate, legally she signed over her rights to him’, and therefore … and they went, ‘Well sorry, you know, this is … you don’t have an option on it’, and I went well… ‘Legally I’m his mother and father.’ They said, ‘Just fill out the information twice’, because most data entry systems won’t actually say it’s duplication, and that’s how we got past it. (John)

These instances, in which fathers were asked about the identity of their children’s mothers, showcase that doing family (i.e. travelling internationally or accessing health services) sometimes involved open discussions about familial circumstances that led to negotiations about what should be documented. Such negotiations may be more (Andrew) or less (John) explicit (Finch and Mason, 1993). John’s experience of duplicating material (also shared by other fathers in the study) would additionally suggest that in some circumstances, fathers and other social actors are equally aware that official forms may ultimately display ‘families we live by’, rather than ‘families we live with’ (Gillis, 1996). In such cases, questions of what is being documented, to whom and for what purpose, become central.

In addition to duplicating material, fathers described instances in which documents had been completed with inaccurate information. For example, Tom explained how he had listed his ex-partner on the birth certificate of his son, because he was born before single fathers could be solely listed on this document in the UK, and that he had been registered for egg donation treatment as part of a heterosexual couple with the woman acting as his surrogate (‘In hindsight, they thought we were a couple, which we weren’t’). On one level, these may be seen as ‘unsuccessful displays’: attempts to display family that do not ‘work’ (i.e. are not recognised by others as family-like; Gabb, 2011). Yet intentionally fictionalising or otherwise not challenging others’ assumptions in ‘official’ displays may also reveal something important about the meanings different actors afford to documents, and the varied purposes they serve in doing family relationships. For fathers such as Tom, legal and institutional documents appear to be a means through which family relationships are enabled to ‘work’ without obstruction. The content of documents is, in such cases, subordinate to their function.

This relates to a broader point: the meanings and functions of the documents of family life are not fixed. As we have seen, documenting involves processes of negotiation that are in most cases ‘tightly constrained’ (Finch and Mason, 1993). Yet these examples, in which documents function to enable family practices, would suggest that different types of negotiation – including those of a silent nature – also take place. Different fathers may engage with different documents in different ways, or similar documents in different ways in different contexts. For instance, Andrew, who was quoted above in negotiation with airport security officials regarding his children’s birth certificates, also explained that he ‘had to break the law and lie about being gay’ on other official documentation, having become a parent when it was illegal in his US state for gay men to adopt. Together, these examples attest to the conceptual contribution of documenting to the literature on displaying and doing family in capturing the tensions between institutional parameters and individual family practices, and how these are contextually negotiated.

### Documenting as Creating and Sustaining Family Legacies

Building upon Finch’s (2007: 78) understanding of display through narratives, the ‘stories people tell to themselves and to others about their own family relationships’, documenting as creating and sustaining family legacies emphasises the ‘testimonial’ or ‘story-like’ nature of some of the documentary forms of paper-work also undertaken by fathers. It is noteworthy that these documents included those created by fathers themselves, as in websites on which fathers documented their journeys to parenthood and their experiences as parents. Although the intended audience of these sites was not made explicit, some fathers advised that we review them as part of our research, suggesting that they were perhaps intended as family testimonies. Given that several fathers described having used online resources to research becoming a parent without a partner, it is also likely that the websites were intentional displays to other single men that their family ‘works’.

Some fathers used other – institutional – forms of documentation in this process, notably in terms of media appearances and participation in academic research. For Jared, this meant that, ‘in many cases people, before I’ve met them have heard about me or read about me’. Similarly, Alex described being ‘known on the grapevine … for having done it, because it’s still quite rare’. It is noteworthy that these fathers had had their children, now in infancy, through surrogacy. Yet Joseph, an adoptive parent to a teenager, also explained that ‘we’ve been in the local newspaper, we’ve been on TV, we’ve been in the *New York Times*’, later saying of his family that, ‘our entire life is kind of an activist position’. Joseph also emphasised his involvement in academic research in a terms that would suggest its legacy function:

I think almost all of us realise that it’s important to get the research out there because otherwise if we don’t have research people will continue to tell lies about our lives and our families and our children, which we can’t stop.

Participating in research thus appeared for Joseph to be a form of paper-work that enabled him to implicitly challenge normative definitions of ‘the family’, and one that had the potential to ensure that his family legacy would be sustained over time (Golombok, 2020). Similarly, Frank described that simply ‘living’ good parenting (Smart and Neale, 1999) was work against normative assumptions about what families should look like (‘I knew a lot of people were watching what I was doing and so I knew that it was important to do the job just not right, but extra right’). These examples attest to documenting as creating and sustaining not only individual family legacies but also legacies about family lives as they are lived within specific minoritised communities. Notably, these examples also reveal feelings of scrutiny among fathers – both among those who became parents several decades ago, and those whose children are currently infants – suggesting that the ‘involved’ father (Dermott, 2008) is not only ideological, but also ideologically restricted to fathers who do family in specific ways (i.e. with female partners).

While these fathers’ activities would suggest that documents with a legacy function simultaneously function to challenge social norms, other fathers used documents to create and sustain family narratives that rather minimised their difference to others. Asif, for example, recorded that he had been widowed on the membership form for his local community group:

We go to this meet-up group for people who are divorced with kids … But I just wrote on the form … I just wrote that I was widowed … I just didn’t really want to get into this whole surrogacy thing with like … I didn’t want them to all think I was gay and be the different one. You don’t know how they’re going to react. (Asif)

Asif thus described documenting as an activity undertaken with potentially negative social responses in mind. It is notable that in his interview, Asif also described having created a website about his journey to fatherhood, reinforcing the point that documenting is a process that is situationally driven, and one that may involve different displays across contexts and over time. As in previous analyses of ‘passing’ (Bower-Brown and Zadeh, 2021), Asif’s account about completing the community group membership form nonetheless indicates the ubiquitous, everyday nature of paper-work. Indeed, across these examples, we see that the work of doing ‘non-normative’ family relationships does not end once fathers gain legal recognition, irrespective of whether and how they choose to conform to, or contest, ‘the family’ in their daily practices. Geoff, for instance, shared Asif’s concerns that he may receive undesirable social feedback, yet described having emailed his extended family as follows:

I wanted to just clarify how I am becoming a parent without being in a relationship, or without being married or whatever, and some of them are religious and all that so … I just didn’t want speculation or people feeling like it was taboo to ask, so I wanted to just put it out there, again as part of that planning for [child] to arrive in a place where the air is clear, so that was, yeah, so that was a big … there was an email that went to a lot of people in my family where I just announced the arrival of [child] but also kind of explaining a little more of the background.

Beyond being a simple birth announcement, a practice common to many families, Geoff used this opportunity to tell the story of his family’s origins. That his narrative was documented was explicitly described as a means of avoiding becoming the subject of gossip, and/or being subject to misrecognition (Almack, 2008; Finch, 2011). Yet other such family-directed displays appeared to function less as a means of avoiding stigma and more to narrativise family experiences. As found in our research with single mothers (Zadeh, 2020), fathers commonly documented their paths to parenthood using homemade story-books, in anticipation of future conversations with their child(ren) about this. John had continued to add to his book after his son’s birth. Describing this, he illustrated that his family display was self- as well as other-directed, thus exemplifying Finch’s (2007) description of narratives as stories that people tell to themselves as well as to others:

Yes, he’s got the little booklet but being able to actually see all of this and … even for me to actually have a look through and go, ‘Oh my god, you’ve changed so much.’ So it’s … yeah, the books are as much for me as for him. (John)

## Conclusion

In this article, we sought to develop the notion of documenting as an extension of the literature on doing and displaying families. We explained that this concept is the result of working through empirical material from our research with single fathers. While family display (Finch, 2007) has great purchase in making sense of much of this material, we developed the concept of documenting to explain the interrelationships, and tensions, between the institutional parameters and individual practices of displaying family evident in our research. In so doing, we have inserted into a literature that deliberately distanced itself from a priori definitions of ‘the family’ (Morgan, 1996, 2011) an emphasis on the role of bureaucratic understandings of family life: that is, on fatherhood, as well as fathering (Morgan, 2004). This has allowed us to conceptualise our findings without either relinquishing or otherwise dramatically recasting the notion of family display.

Beyond clarifying our findings, what – if anything – does documenting add to the conceptual toolkit of family practices? As we have defined it, documenting refers to the official and unofficial processes of producing material in displaying or doing family, and it is a concept that *contains within itself* the tensions between institutional and individual meanings. This is because families the world over require documentation, yet this process often involves ideas about ‘the family’ that do not chime with contemporary relationships and experiences (Gibson, 2016; Nordqvist, 2012). As our empirical material has shown, documenting involves negotiations between different social actors who are generally unequal in terms of their authority and agency to impose situational meaning. Such negotiations are as much about pieces of paper as they are about deeply rooted social norms, which are often embedded in policy, regarding how to define and regulate family relationships. This does not mean that such relationships cannot be done if not documented (see, for example, Weeks et al., 2001), but rather makes the point that to theorise how ‘non-conventional’ relationships are done and displayed, particularly in relation to institutional and social constraints, additional concepts (of which documenting may be one) are helpful.

Our research has shown that documenting as a practice serves multiple functions in how single fathers do and display family to different audiences. Our examples are almost entirely about written material, perhaps because the main way in which fathers ‘met’ institutions was in relation to texts. The concept therefore brings with it a renewed emphasis on the material means of doing family, as in Finch and Mason’s (2000) research on the role of keepsakes and heirlooms in family inheritance practices, and Holmes’ (2019) work on the ‘passing on’ of mundane objects in everyday family relationships. Whether or not documenting can be conceptually extended to include these formats without relinquishing its meaning requires empirical exploration, though it seems probable that the concept should exclude types of display without material form (e.g. oral traditions, narratives). This is, first, because physical documents often featured in fathers’ accounts, seemingly conferring a kind of symbolic capital (Bourdieu, 1986) that could not be realised without them. Second, some fathers described writing the family down with feelings such as fatigue, frustration, hope and surprise, suggesting that material documents may have a particular affective resonance that does not manifest in quite the same way in non-material forms of display.

We have conceptualised documenting as *paper-work* to emphasise the labour (emotional and otherwise) undertaken by those in ‘non-conventional’ relationships to do and display family in contexts that are overwhelmingly unaccommodating of them. As our initial example from Joseph – who spoke about ‘fill[ing] out all these papers over and over and over and over again’ – attests, this paper-work is often repetitive, and sometimes, circular. Paper-work is thus conceptually reminiscent of Ahmed’s (2019) ‘wall work’, a term used to evoke the image of banging one’s head against a wall to describe working within institutions on matters of diversity. Paper-work is similarly an everyday practice that reflects inequities in social actors’ authority and agency. Importantly, this work is required of fathers despite their generally relatively privileged socio-economic status (Harman and Cappellini, 2015): in fact, it may, paradoxically, only be available to them because of it. But while this process enables some fathers to authenticate their family relationships by meeting the ‘official’ requirements of display, it is also the means through which they do family-like things, and that which aids them in conversations with their children about why some families look like theirs, and others look like ‘the family’. As per Heaphy’s (2011) insights on display, documenting is therefore neither entirely constraining nor creative – it is both.

We have suggested that documenting is a concept that is brought to life in studies of what Finch (2007) originally referred to as ‘non-conventional’ family relationships. Like display, practices of documenting may also be relevant, but are likely not notable, to those in ‘normative families’ (Almack, 2008). Our focus has been on single fathers, and it has mostly concerned written documents described to us in oral interviews. The concept should now be extended to other empirical work, so that it may be further developed.
